# PbS Quantum Dots Decorating TiO_2_ Nanocrystals: Synthesis, Topology, and Optical Properties of the Colloidal Hybrid Architecture

**DOI:** 10.3390/molecules25122939

**Published:** 2020-06-26

**Authors:** Carlo Nazareno Dibenedetto, Teresa Sibillano, Rosaria Brescia, Mirko Prato, Leonardo Triggiani, Cinzia Giannini, Annamaria Panniello, Michela Corricelli, Roberto Comparelli, Chiara Ingrosso, Nicoletta Depalo, Angela Agostiano, Maria Lucia Curri, Marinella Striccoli, Elisabetta Fanizza

**Affiliations:** 1 Dipartimento di Chimica, Università degli Studi di Bari, Via Orabona 4, 70126 Bari, Italy; carlo.dibenedetto@uniba.it (C.N.D); michela.corricelli@gmail.com (M.C.); angela.agostiano@uniba.it (A.A.); marialucia.curri@uniba.it (M.L.C.); 2 CNR-Istituto per i Processi chimico Fisici (CNR-IPCF), SS Bari, Via Orabona 4, 70126 Bari, Italy; l.triggiani@ba.ipcf.cnr.it (L.T.); a.panniello@ba.ipcf.cnr.it (A.P.); r.comparelli@ba.ipcf.cnr.it (R.C.); c.ingrosso@ba.ipcf.cnr.it (C.I.) n.depalo@ba.ipcf.cnr.it (N.D.); 3 CNR-Istituto di Cristallografia (CNR-IC, Via Amendola, 122/O, 70126 Bari, Italy; teresa.sibillano@ic.cnr.it (T.S.); cinzia.giannini@ic.cnr.it (C.G.); 4 IIT- Istituto Italiano di Tecnologia, via Morego 30, 16163 Genova, Italy; Rosaria.Brescia@iit.it (R.B.); Mirko.Prato@iit.it (M.P.)

**Keywords:** colloidal heterostructures, seed mediated growth, heterogeneous nucleation, PbS/TiO_2_ heterostructure, TiO_2_ nanocrystal defects

## Abstract

Fabrication of heterostructures by merging two or more materials in a single object. The domains at the nanoscale represent a viable strategy to purposely address materials’ properties for applications in several fields such as catalysis, biomedicine, and energy conversion. In this case, solution-phase seeded growth and the hot-injection method are ingeniously combined to fabricate TiO_2_/PbS heterostructures. The interest in such hybrid nanostructures arises from their absorption properties that make them advantageous candidates as solar cell materials for more efficient solar light harvesting and improved light conversion. Due to the strong lattice mismatch between TiO_2_ and PbS, the yield of the hybrid structure and the control over its properties are challenging. In this study, a systematic investigation of the heterostructure synthesis as a function of the experimental conditions (such as seeds’ surface chemistry, reaction temperature, and precursor concentration), its topology, structural properties, and optical properties are carried out. The morphological and chemical characterizations confirm the formation of small dots of PbS by decorating the oleylamine surface capped TiO_2_ nanocrystals under temperature control. Remarkably, structural characterization points out that the formation of heterostructures is accompanied by modification of the crystallinity of the TiO_2_ domain, which is mainly ascribed to lattice distortion. This result is also confirmed by photoluminescence spectroscopy, which shows intense emission in the visible range. This originated from self-trapped excitons, defects, and trap emissive states.

## 1. Introduction

The colloidal approach, which is extensively used to prepare nanoparticles (NPs) and nanocrystals (NCs) with a variety of sizes and shapes [1,2,3,4], has, in recent decades, made a step forward by fabricating hybrid NPs [5,6,7,8,9,10,11] based on the combination of two or more materials in one solid nano-object for application in several fields, including biomedicine, environment, catalysis, and sensing [12,13,14,15,16]. In principle, any desired inorganic material can be purposely assembled to form the hybrid structures, characterized by properties that possibly derive from a simple combination, enhancement, or mitigation of the properties of the individual materials or can bring on completely new physical and chemical properties. Metal-metal [5,6], semiconductor-semiconductor [17,18,19,20,21,22,23], metal-semiconductor [24,25,26,27], and magnetic-semiconductor [28,29] hybrid structures have been prepared by different solution-phase approaches such as seed mediated epitaxial and non-epitaxial deposition [14,17,30,31,32,33], cation exchange [34,35,36], and coupling promoted by the bifunctional linker [37,38]. With the exception of the last strategy, where pre-synthesized NPs are assembled ex-situ at distances inherently induced by the linker, the other approaches rely on the growth of the new phase on pre-existing NPs with the two domains in intimate contact. Face miscibility and lattice mismatch between the two phases control the feasibility of the hybrid structure and its final topology. Cation exchange, which consists of the kinetically driven replacement of cations of pre-existing NCs with new cations while retaining anion sub-lattice, has been exploited for the fabrication of multi-component materials under a mild temperature condition [34,35]. The new phase, nucleated at the NC surface facet, may topotaxially grow toward the interior of the NCs or form original morphologies due to facet-dependent exchange reaction, nanoscale asymmetry, and anisotropy. Although this strategy shows great potentiality for the easy fabrication of novel heterostructures, it has been mainly limited to the fabrication of hybrid structures belonging to the II–VI, I−III−VI, and IV−VI classes of semiconductors and characterized by the same anion for both domains. Conversely, a huge variety of hybrid structures with each component having a different size, shape, spatial orientation, composition, and crystalline structure, have been synthesized by the multi-step seed-mediated growth [10,39,40,41]. The reaction scheme consists of the first step of synthesis of the seeds, which is followed by the heterogeneous nucleation and growth of the new phase upon precursor’s injection. Even though the presence of seeds in solution provide a thermodynamic and kinetic gain to the nucleation of a new phase by References [8,9,42], and, even though heterogeneous nucleation results in more favoured than homogenous nucleation, the achievement of a high yield of hybrid structures remains challenging, which is often affected by the separate homogenous nucleation of the new phase. According to the colloidal synthesis theory, the Gibbs energy barrier to heterogeneous nucleation is lower than that of homogenous nucleation due to a series of factors including wettability between seeds and nuclei, and further decreases at an increase of a new phase monomer supersaturation and reaction temperature. Similarly, the presence of the seeds affects the nucleation rate. In spite of the fact that heterogeneous and homogeneous nucleation paths follow the same kinetic law, heterogeneous nucleation is generally faster than homogeneous nucleation under an actual supersaturation condition. However, while at low supersaturation, heterogeneous nucleation dominates at high supersaturation. Homogenous nucleation has been reported to be the preferred path [43]. Though temperature and supersaturation define the synthetic condition that make the formation of hybrid structures prevail over homogeneous nucleation, the final hybrid material topology is mainly shaped by the interfacial strain and facet dependent on chemical reactivity [9,23]. A positive strain energy term (γ_strain_) contributes to the interfacial tension (γ_i_) and, together with the solid/solution surface tension of seeds (γ_seed/solution_) and nuclei (γ_nuclei/solution_), defines the overall surface tension (Δγ = γ_nuclei/solution_ + γ_i_ – γ_seed/solution_). The Δγ value allows prediction of the growth mode of the heterostructure as well as the topology of the hybrid NP. Centrosymmetric core-shell hybrid structures form when the lattice constants of the two components do not differ significantly (Frank-van der Merwe mode, Δγ < 0), while non-centrosymmetric structures are achieved at Δγ < 0, only if interfacial energy between the two materials is large enough and when certain regions on the seed surface are accessible and reactive. At Δγ > 0 and at high lattice mismatch (> 5%) between the two phases, the deposition of the new nuclei on the seed occurs by formation of islands-like structures (Volber-Weber mode). In this case, the energy strain is released by atoms rearranging or by interfacial misfit dislocation. However, these theoretical predictions do not take into account the presence in solution phase strategies of organic surfactants, which modulate the solid/solution interfacial tension and enable the formation of heterostructures, even combining dissimilar materials [20].

This work focuses on the synthesis of hybrid nanostructures based on the nucleation of PbS NCs onTiO_2_ NPs. Colloidal TiO_2_/PbS hybrid NCs have been prepared following diverse strategies, such as seed-mediated hot-injection [20], ultrasound ionic deposition [44], successive ionic layer adsorption, and a reaction method (SILAR) [45], or by PbS and TiO_2_ NCs assembling with very short bifunctional linker molecules [38]. Such a type of hybrid structure has gained increasing attention in recent decades for its potential application in solar cell devices [46,47,48]. Coupling of the TiO_2_ wide band gap semiconductor (3.2 eV) with PbS NPs with a size dependent narrow band gap that extends to the NIR region may overcome the intrinsic limitation of TiO_2_, which only absorbs the ultraviolet portion of the solar spectrum [49,50] thanks to PbS-mediated sensitization process [51,52]. Enhanced solar light harvesting and efficient electron transfer have been reported to improve the performances of solar cells [20,44]. However, charge carrier dynamic at the interface strongly depends on the appropriate choice of the two material band offsets. Quantum confined PbS NCs, characterized by a large exciton Bohr radius [44], offer the possibility to well control the band edge alignment with TiO_2_ by simply tuning their size. Solar light photogenerated electron/hole pairs can be forced either to recombine within the PbS materials, according to type-I heterojunction behaviors or to separate by efficient interparticle electron transfer in a type-II heterojunction [8]. However, as pointed out by Trejo et al. [53], the deposition of PbS over TiO_2_ suffers from the large lattice mismatch between the two materials. Island-like structures of PbS, grown by chemical vapour deposition on crystalline TiO_2_ film, show a PbS domain characterized by bond distortion from the bulky crystalline phase, which results in changes in the electronic structure of the PbS and increase of the energy gap. Similarly, as described by Acharya et al. [20], the final topology of the TiO_2_ nanorods/PbS hybrid structure, grown by the solution phase strategy, and the PbS domain size are dictated by the large interfacial strain at the two-material interface. The work pointed out that, thanks to the use of colloidal NCs as seeds in the synthesis, the interfacial strain can be tuned through the TiO_2_ ligands, which results in near-epitaxial small island-like or single large PbS structures deposited on TiO_2_ nanorods [20]. The growth mode and the PbS domain size, lastly, defines the electron transfer properties at the hybrid structure interface [20]. This results in a type-II hybrid heterojunction and efficient electron transfer from PbS to TiO_2_ domains [20], when PbS domains with sizes below 7 nm decorate the TiO_2_ nanorods. 

The present study aims at synthesizing and extensively characterizing TiO_2_/PbS hybrid NCs prepared by a seeded growth approach using purposely functionalized TiO_2_ NCs as seeds and allows the PbS domains growth, in situ, by the hot injection method. The work wants to examine the critical role of interfacial strain in the fabrication of such hybrid TiO_2_/PbS NCs in directing the heterostructure topology, domain structure, and final optical properties. The effectiveness of PbS deposition on TiO_2_ seeds, passivated with a native layer of oleic acid (OA), or surface modified with oleylamine (Olam), and the final hybrid structure topology, have been explored by varying the precursor concentration, and, thus, monomer supersaturation as well as injection temperature. The systematic morphological investigation allows defining the synthetic conditions, which brings a high yield of hybrid nanostructures under limited homogeneous nucleation, successfully attained at specific precursor concentration and under controlled temperature conditions, when using Olam-capped TiO_2_ NCs as seeds. It has been shown that the presence of Olam as a capping agent, loosely bound at the TiO_2_ NCs surface, allows an easier PbS monomer deposition, which provides the hybrid structure formation. Lastly, the surface modification and interfacial strain between TiO_2_ and PbS have been found to strongly affect the structural and optical properties of the hybrid NPs and, in particular, the optical properties of the TiO_2_ domain. 

## 2. Results and Discussion 

**Figure 1:**
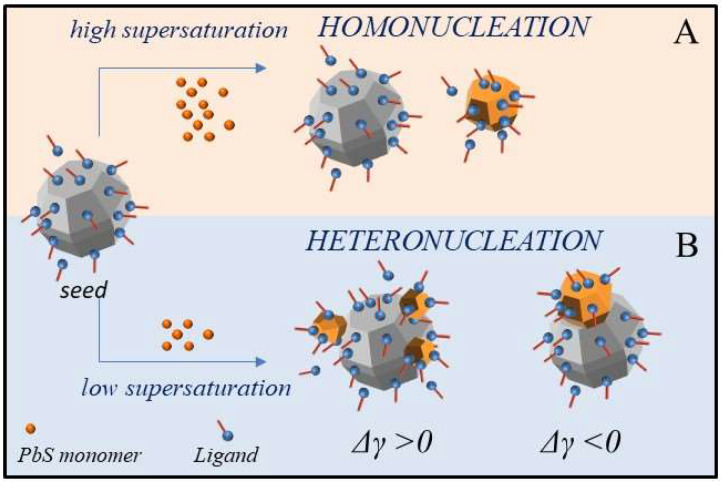
Seeded-growth under high (**A**) and low (**B**) supersaturation condition. Homogenous nucleation represents the upper-limit to heterogeneous nucleation. At low supersaturation, the overall interfacial energy (Δγ = γ_nuclei/solution_ + γ_i_ – γ_seed/solution_, with γ_i_ = interfacial tension) defines the topology of the hybrid structure that, under high interfacial strain, may result in segregated phases (Δγ < 0) or island-like structures (Δγ > 0, Volmer-Weber growth mode). The grey dodecahedral shape NP represents the TiO_2_ NC seeds while the orange structures represent the PbS NCs.

In this scenario, the synthesis of TiO_2_/PbS hybrid NCs by seeded-mediated growth and hot injection approaches has been developed. The main aim of this work is to examine how parameters such as injection temperature, precursor concentration, supersaturation, and ligands composition at the TiO_2_ seed surface can modulate the energetic and kinetic factors that promote colloidal heterogeneous nucleation over homogeneous nucleation, and determine the yield and topology of the heterostructures. The general procedure relies on the hot-injection [54] at different temperatures ranging from 120 °C to 80 °C, into the TiO_2_ seeds suspension, of the Pb-oleate precursor, prepared by decomposing PbO in oleic acid and ODE. This is followed by the addition of HMDS solution in ODE (Pb:HMDS molar ratio 5:1). Upon injection, Pb-oleate and HMDS readily decompose, which results in the sudden release of monomers that burst the nucleation of PbS [55,56,57,58]. Homogenous nucleation represents the upper-limit to heterogeneous nucleation under a high supersaturation condition (Figure 1A), while, at low supersaturation (Figure 1B), the interplay between the lattice mismatch and the solid/solution surface tension for each phase defines the topology of the hybrid structure. In addition, increasing the injection temperature makes the exchange dynamic faster between surfactants bound at the seeds’ surface and the free ligands in solution, which renders the seed surface more accessible to the monomer deposition, and, concomitantly, allows faster monomer diffusion [29], which profitably improves heterogeneous nucleation. However, in general, above a certain temperature threshold, which depends on the reaction mixture composition and precursor nature, the higher the temperature, the faster becomes the homogeneous nucleation. In this case, two precursor concentrations have been used with [Pb^2+^] ranging from 0.01 M to 0.005 M and [HMDS] from 0.002 M and 0.001 M and decreasing injection temperature, namely 120 °C, 100 °C, and 80 °C, have been investigated by fixing the growth temperature at 80 °C. 

The reaction has been stopped after 10 min since prolonged heating has been observed to be always accompanied by the reaction mixture discoloration, which is likely ascribed to partial dissolution of the PbS domains.

### 2.1. Fabrication of TiO_2_/PbS Hybrid NCs 

**Figure 2:**
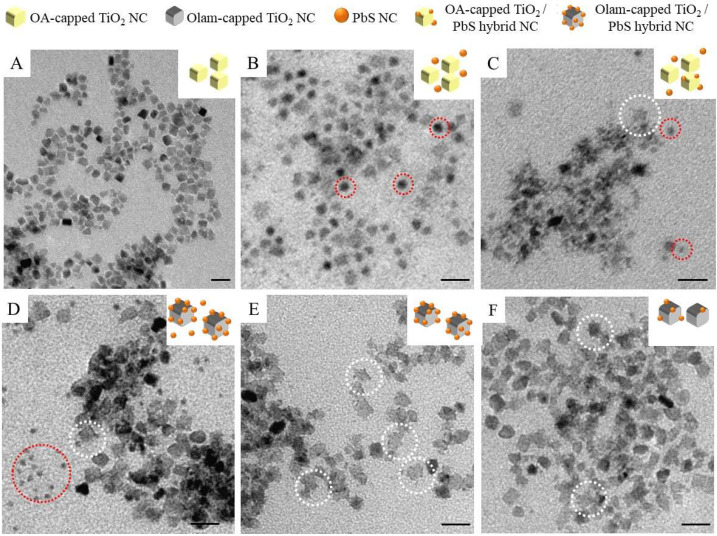
TEM micrographs (scale bar 20 nm) of oleic acid (OA)-capped TiO_2_ NCs before (**A**) and after (B-C) injection of (**B**) [Pb^2+^] = 0.01M, [HMDS] = 0.002 M at 120 °C and (**C**) [Pb^2+^] = 0.005M, [HMDS] = 0.001 M at 100 °C. (**D**–**F**) oleylamine (Olam)-capped TiO_2_ NCs after injection of [Pb^2+^] = 0.005M, [HMDS] = 0.001 M at 120 °C (**D**), 100 °C (**E**), and 80 °C (**F**). Dashed red and white circles in the picture used to highlight homogenously nucleated PbS NCs and TiO_2_/PbS hybrid NCs, respectively.

**Figure 3:**
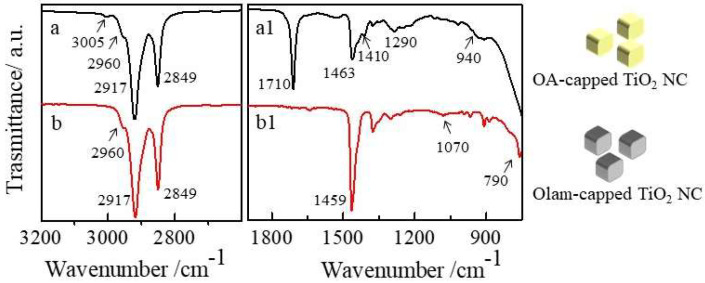
FTIR spectra in the ATR mode of oleic acid (OA)-capped (**a**–**a1**) and oleylamine (Olam)-capped TiO_2_ NCs (**b**–**b1**).

Monodispersed platelet-like colloidal TiO_2_ NCs seeds with an average size of nearly 7 nm (σ% = 13%, Figure 2A) have been synthesized by an alcholisis reaction between OA and 1-octadecanol that results in water release in the reaction flask [59] and titanium ethoxide hydrolysis, which is followed by the titanol condensation reaction. The FTIR spectrum of the TiO_2_ NC sample (Figure 3a and Figure 3(a1) allows disclosing the ligand composition and the type of ligand coordination at the NC surface. The 3300–2800 cm^−1^ range (Figure 3a) shows the vibrational modes of the *cis*-9-octadecenoil hydrocarbon chain of OA (see Figure S1A) used as a coordinating agent during the synthesis with characteristic absorption peaks at 2917 cm^−1^ and 2849 cm^−1^, ascribed to the intense symmetric and antisymmetric C-H stretching of methylenic groups at 2960 cm^−1^, attributed to the antisymmetric stretching of the terminal -CH_3_ group, and, at 3005 cm^−1^, corresponding to the weak but definite band, which is characteristic of the =C-H symmetric stretching.

The peaks at 1463, 1410, 1290, and 940 cm^−1^ (Figure 3(a1)) can be ascribed to –CH_2_– bending of the hydrocarbons chain, –C–O–H in-plane bending, –C–OH stretching vibrations, and –O–H out of the plane mode of the carboxylic acid moiety, respectively. The presence of an intense –C=O stretching mode at 1710 cm^−1^ confirms that OA binds the TiO_2_ NCs surface through the oxygen of the carbonyl group in a monodentate R–C(=O)–O form rather than forming bidentate RCO_2_-M structures [60,61,62]. The FTIR spectrum also shows the characteristic broad and intense stretching of metal–oxygen TiO_2_ bonds below 800 cm^–1^. The as-prepared TiO_2_ NCs have been addressed in the paper as OA-capped TiO_2_ NCs.

The first set of reactions for the synthesis of hybrid TiO_2_/PbS NCs has been performed by using seeds as the OA-capped TiO_2_ NCs suspensions. TEM micrograph of the sample, prepared upon injection at 120 °C of Pb-oleate at [Pb^2+^] = 0.01M and HDMS at 0.002M is reported in Figure 2B. Small dots of nearly 5 nm in diameter (σ% = 14%), highlighted by the dashed red circles in the picture, are visible together with features clearly ascribed to TiO_2_ NCs (Figure 2A). Furthermore, isotropic growth and formation of a spherical PbS domain are typically achieved since OA is the sole ligand in the reaction mixture released upon Pb-oleate precursor decomposition and/or desorbed from the TiO_2_ surface at a high temperature, and, given that OA does not preferentially bind any specific PbS face, and dynamically desorbs and adsorbs at the PbS surface [54].

Homogeneous nucleation of PbS has not been found to be suppressed even by decreasing the injection temperature at 100 °C (Figure S2, [HDMS] = 0.002M, [Pb^2+^] = 0.01M), which would have been expected to favor heterogeneous nucleation over homogeneous nucleation nor by decreasing the precursor concentration (Figure 2C, [HMDS] = 0.001M, [Pb^2+^] = 0.005M) while keeping the injection temperature constant. However, in the latter case, even to a lower extent, morphologies characterized by tiny dots decorating the TiO_2_ NCs seeds have been detected (white dashed circle in Figure 2C), which confirms that depletion of the monomer under low supersaturation condition may improve the yield of heterostructures. The monomer concentration used for the synthesis of the sample reported in Figure 2C has been applied for further experiments.

To optimize reaction conditions toward high yield formation of TiO_2_/PbS heterostructures without homogeneous nucleation, it should be considered that hybrid structures are difficult to achieve. In fact, since a high energy strain [20,53] has been reported between PbS and TiO_2_, this, in principle, would not favor the deposition of PbS in intimate contact with the TiO_2_ domains. However, TiO_2_/PbS heterostructures have been reported to be successfully achieved when TiO_2_ {001} facets merge with the cubic PbS (rock-salt) {100} face, even though the two faces show a substantial 6.9% lattice mismatch [20,53]. In this case, OA ligands, which are known to specifically tightly bind to the TiO_2_ {001} faces [63,64], contribute in limiting the formation of the hybrid structure, which hinders the deposition of PbS on the seed surface. A different scenario is, then, expected by replacing OA with a new capping layer that less strongly passivates the TiO_2_ NCs {001} faces. After extensive purification steps of the TiO_2_, NCs to partially remove the physisorbed OA and the excess of OA in solution [65], Olam has been added in large excess for the exchange reaction. Olam is an l-type ligand and it loosely binds by means of nitrogen electron pair the TiO_2_ NCs [66], preferentially coordinating the {101} faces, which leaves the {001} faces that are highly reactive and available for PbS deposition. According to the ligand-exchange rules [66], since OA binds the TiO_2_ NC surface through the monodentate oxygen of the carboxylic moieties, acting as an l-type ligand, it can be effectively exchanged by Olam without affecting the electroneutrality of the TiO_2_ NCs [66]. The FTIR spectrum of the Olam treated TiO_2_ NCs is reported in Figure 3b and 3(b1). The spectral range between 3100 cm^−1^ and 2800 cm^−1^, characteristic of the symmetric and antisymmetric C-H stretching modes of saturated and unsaturated hydrocarbons, does not show any appreciable change if compared to the untreated OA-capped TiO_2_ NCs, since both OA and Olam bring the same alkyl (*cis*-9 octadecenoyl) chain. However, the inspection of 1900–750 cm^−1^ wavenumber range (Figure 3(b1)) shows the complete disappearance of the peaks at 1710 cm^−1^ (*υ*_s_ –C=O) and at 940 cm^−1^ (O-H out of plane modes) characteristics of the carboxylic group, which confirms the removal of the OA from the TiO_2_ NC surface. Even though addition of Olam can promote the formation of oleyl ammonium-oleate salt due to acid-base equilibrium, the two bands’ characteristics of oleate, centered at 1528 and 1442 cm^–1^, respectively, are not detected. The FTIR spectrum of the 1700–800 cm^−1^ region, reported in Figure 3(b1), mainly dominated by the intense peak at 1459 cm^−1^ ascribed to the –CH_2_ scissoring, shows a peak at 1070 cm^−1^ ascribed to the C–N stretching mode, and a peak at 790 cm^−1^ ascribed to the –NH_2_ wagging, superimposed to the strong broad stretching of metal–oxygen bonds below 800 cm^–1^, which is characteristic of the TiO_2_ NC samples. These active stretching modes and the lack of the peak at 1616 cm^−1^, which corresponds to the –NH_2_ scissoring, is visible in the spectrum of the free Olam (Figure S1(B1)). This suggests that Olam coordinates the surface of the TiO_2_ NCs through the amino groups.

The Olam-capped TiO_2_ NCs have been used as seeds for preparing a new set of samples. Figure 2D–F reports the morphological characterization of the samples obtained at [HMDS] = 0.001 M, [Pb^2+^] = 0.005 M, and at an injection temperature of 120 °C (Figure 2D), 100 °C (Figure 2E), and 80 °C (Figure 2F). A high yield (white dashed circle Figure 2D) of TiO_2_ NCs, decorated with tiny dots (size nearly 1.8 nm and σ_%_ = 14%), ascribed to TiO_2_/PbS NCs, are displayed in Figure 2D, which corresponds to the sample prepared at an injection temperature of 120 °C, even though isolated dots of nearly 2.9 nm (σ_%_ = 9%) are also visible (red dashed circle Figure 2D), which suggests the concomitant occurrence of both homogeneous and heterogeneous nucleation. The decrease of the precursors’ injection temperature down to 100 °C (Figure 2E) mainly results in sole nanostructures, based on TiO_2_ NCs decorated with small spots (nearly 1.7 nm and σ_%_ = 13%) and a negligible number of isolated dots. Further reduction of the injection temperature at 80 °C causes only a slight darkening of the solution color upon precursors injection. The TEM micrograph (Figure 2F) reveals a low yield of TiO_2_-decorated structures. Therefore, while in the presence of OA-capped TiO_2_ seeds, the homogeneous nucleation of PbS could not be eluded, even at the lowest tested supersaturation condition. The exchange of OA with Olam has promoted the formation of structures attributed to heterogeneous NCs and the formation of isolated PbS NCs has been strongly limited by decreasing the reaction temperature. Further chemical, structural, and spectroscopic characterization has been carried out for the sample prepared starting from Olam-capped TiO_2_ NCs and injection of PbS precursors ([HMDS] = 0.001 M, [Pb^2+^] = 0.005 M) at 100 °C that shows, by TEM characterization, the limited homogenous nucleation and the higher yield of tiny dots decorated TiO_2_ NCs.

### 2.2. Structure and Chemical Composition of TiO_2_/PbS Hybrid NCs 

**Figure 4:**
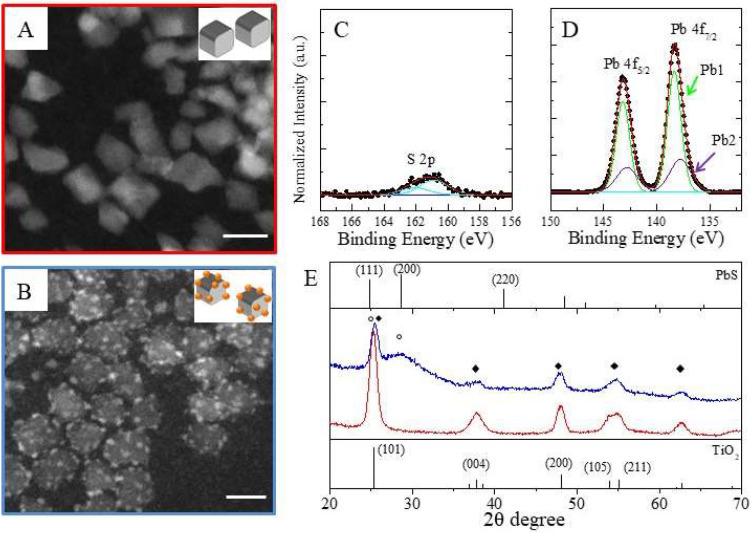
HAADF-STEM images (scale bar 10 nm) of (**A**) TiO_2_ seed NCs and (**B**) TiO_2_/PbS hybrid nanostructures. (**C**,**D**) XPS spectrum of TiO_2_/PbS hybrid NCs in the 130–170 eV range: raw data (scattered plot) and fitting curve of the S 2p (C, light blue line) and of the Pb 4f (**D**), based on two contributions Pb1 (D, green line) and Pb2 (D, violet line). (**E**) XRD spectra of TiO_2_ NCs (red line) and TiO_2_/PbS hybrid (blue line) together with the Bragg hkl reflections positions for TiO_2_ anatase (bottom markers, crystal system: tetragonal, PDF2-ICDD code: 842186) and PbS (upper panel, crystal system: cubic, PDF2-ICDD code: 00-005-0592) crystal structures. The XRD spectra of the two samples are reported, and shifted for the sake of clarity. Filled square and empty circle symbols are ascribed to TiO_2_ anatase and PbS peaks, respectively.

Olam-capped TiO_2_ NCs (Figure 4A) and the TiO_2_/PbS hybrid NC sample (Figure 4B) have been investigated by high-angle annular dark-field-scanning TEM (HAADF-STEM). Based on incoherently scattered electrons, the HAADF-STEM («Z-contrast») imaging mode shows a contrast related to the average atomic number (~Z^1.7^). This translates into high sensitivity to variations in the atomic number of atoms in the sample and, thus, enables an easy identification of areas characterized by a different mean atomic number.

Small bright dots, ascribed to a high mean atomic number material, decorate less bright NCs (Figure 4B), which corresponds to TiO_2_ seeds (Figure 4A). The observed island-like structure of the material decorating the TiO_2_ NCs retraces the Volmer-Weber growth mode, as expected, from the high lattice mismatch between PbS and TiO_2_. STEM-EDS analysis (Table 1) and XPS characterization (Figure 4C) have been carried to unveil the chemical composition of the hybrid structure.

The STEM-EDS data confirm the presence Pb and S in the TiO_2_/PbS hybrid NCs, even though the latter content is lower than that expected to be consistent with the presence of PbS domains. However, it must be mentioned that quantification of sulfur in PbS by EDS is affected by a large uncertainty due to the overlap of the S Kα (2.31 keV) and Pb Mα (2.34 keV) X-ray peaks, given the relatively low energy resolution (~100 eV in the mentioned energy range) of EDS. Therefore, the quantification of the S atomic content relies on fitting the two peaks with a consequent uncertainty. Concomitantly, an increase in O content with respect to Ti (O%: Ti% 3.6:1) is revealed in the hybrid NC sample compared to TiO_2_ NC seeds (O%: Ti% 2:1), which is in disagreement with the stoichiometry of TiO_2_. This means that other O sources contribute to the O signal. PbS photo-oxidation [67,68], reasonably taking place in samples stored under air and atmospheric humidity, and residual Pb-oleate, present in excess during the synthesis, can explain the high O content revealed in the hybrid structure. Residual Pb-oleate can also reasonably account for the excess of Pb over S detected by the STEM-EDS analysis. The overall stoichiometry of the TiO_2_/PbS hybrid system has been assessed by XPS characterization (Figure 4C) [68,69]. The XPS spectrum shows the typical peaks of both S (Figure 4C) and Pb (Figure 4D) in the binding energy range of 130–170 eV. In particular, the Pb 4*f* signal is composed of two peaks ascribed to Pb4*f*_7/2_, at 138.4 ± 0.2 eV and Pb 4*f*_5/2_, at 143.2 ± 0.2 eV. The most prominent feature of the Pb4*f*_7/2_ peak position agrees with the presence of Pb ions in the Pb^2+^ oxidation state. This peak has been fitted with two major contributions (Figure 4D), which include a prominent Pb1 component centered at 138.6 eV, correlated to binding energy values of oxidized or hydroxylated species (PbSO_3_, Pb(OH)_2_) or residual Pb-oleate, expected to have their contributions within the window of 138.4–138.6 eV, according to the NIST database [68] and Pb2, the less intense component, located at 137.8 eV, which is a binding energy value corresponding to Pb in PbS (137.8 ± 0.4eV in the NIST database). The S2*p* signal (Figure 4C) has been fitted with a single component located at 160.9 ± 0.3 eV that can be attributed to S bound to Pb (PbS rock-salt type formation, 160.6 ± 0.6 eV in the NIST database) [70]. The quantitative analysis, made by comparing the signal of the Pb component at 137.8 eV and the S, with each one attributed to the PbS, shows a Pb: S ratio of 1:1, which reflects the stoichiometry of bulk-like PbS. Therefore, the results of the overall HAADF-STEM, STEM-EDS, and XPS characterization (Figure 4C) suggest the formation of small PbS dots with residual Pb-oleate and other oxidized Pb species [68,69].

X-ray diffraction patterns of TiO_2_ (Figure 4E red line) and TiO_2_/PbS hybrid NCs (Figure 4E: blue line) together with the Bragg hkl reflections for TiO_2_ anatase and PbS crystal structures are reported in Figure 4E. TiO_2_ NCs show a pattern characterized by peaks that can be indexed with the crystallographic structure of TiO_2_ anatase (crystal system: tetragonal, PDF2-ICDD code: 842186), while the hybrid TiO_2_/PbS NCs shows a pattern profile characterized by the presence of both PbS rock-salt (crystal system: cubic, PDF2-ICDD code: 85128) and TiO_2_ anatase (crystal system: tetragonal, PDF2-ICDD code: 842186) crystalline phases. In particular, the diffraction peak at nearly 2θ = 25.4°, has been indexed as the overlap between the (101) of TiO_2_ and (111) of the PbS. The broad peak at 2θ = 28.6° matches the reflection peak (220) of the PbS rock-salt phase, since their broadening accounted for the very small size of the PbS domain, as confirmed by the TEM characterization and XPS chemical analysis. Diffractions peaks at 2θ~37.9°, 48.0°, and 54.6° corresponding to the (004), (200), and (211) hkl Bragg reflections of the TiO_2_ anatase structure are found both in the hybrid structures and in the TiO_2_ NCs. 

The apparent difference in relative integral intensity of specific diffraction peaks in TiO_2_ NCs compared to TiO_2_/PbS hybrid NCs suggests a possible structural change that have been analyzed in the following. The integral intensity ratio for the TiO_2_ reflections (004)/(200) has a value of 1.20 in TiO_2_ NCs, which decreases to 0.37 in TiO_2_/PbS hybrid NCs. Similarly, the integral intensity ratio (101)/(200) is nearly 3.58 in TiO_2_ NCs and becomes 1.86 for the NCs’ hybrid structures. This latter ratio value should be slightly underestimated, which is the peak (200) at 2θ = 25.4° where the overlap between two contributions arise from the TiO_2_ (101) and the PbS (111) reflections. The intensity ratios show that the (004) and (101) TiO_2_ reflections are less represented with respect to the (200) one in the TiO_2_/PbS hybrid NCs, which indicates a decrease of the crystallinity along the [004] and [101] directions for the TiO_2_/PbS NCs hybrid structure with respect to the [200] one.

Bonding distortions in TiO_2_/PbS hybrid structures have been demonstrated in literature [53] with the periodic bonds at the TiO_2_ surface compromising the crystal structure of PbS during its deposition. In this case, XRD data clearly suggest a structural modification of the TiO_2_ seed in the hybrid structure. In order to accommodate the misfit between the two materials’ lattice parameters and reduce the interfacial strain, partial disruption of the TiO_2_ anatase crystallinity, mainly along the [101] and [004] directions, may occur. However, XRD analysis does not provide any quantitative estimation on the PbS crystalline domain due to the small size of this domain in the hybrid structures. 

### 2.3. Spectroscopic Characterization 

Characterizations of colloidal semiconductor NCs by absorption and emission spectra is essential for monitoring the material optical properties in view of their applications in sensing, energy conversion, and optoelectronic devices. In addition, in the case of TiO_2_ NCs, photoluminescence spectroscopy represents a powerful technique for tracking the evolution of defective states [71]. Since the valence and conduction bands of TiO_2_ are associated with the O-2p and Ti-3d states, respectively, rearrangements of Ti and O atoms can bring modifications in the band edge structures [72].

**Figure 5:**
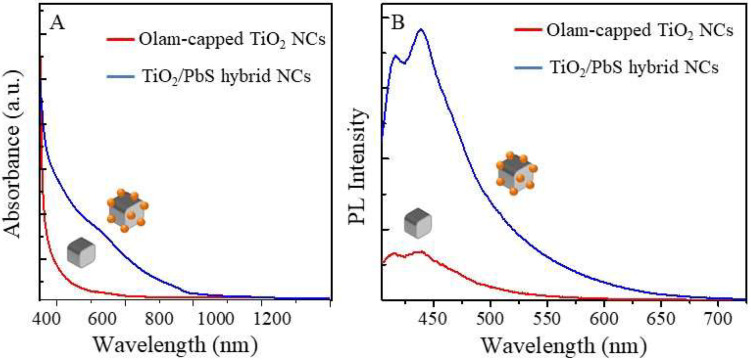
(**A**) UV-Vis-NIR absorbance spectra and (**B**) steady state emission spectra (λ_ex_= 375 nm) in the visible spectral range of the Olam-capped TiO_2_ seeds (red line) and TiO_2_/PbS hybrid structures (blue line). Each sample has been suitably diluted in order to show the same absorbance value at 375 nm.

UV-Vis-NIR absorption spectra (Figure 5A) as well as stationary (Figure 5B, λ_ex_ at 375 nm) and time-resolved emission (Figure S3) in the visible range of Olam-capped TiO_2_ seeds (red line, Figure 5A,B) and TiO_2_/PbS hybrid nanostructures (blue line, Figure 5A,B) have been recorded. TiO_2_ colloidal dispersion (Figure 5A, red line) shows the absorption line profile characteristic of an indirect band gap semiconductor highlighting a steep absorption onset in the UV region, which is consistent with the energy gap of anatase TiO_2_ (3.2 eV), while the absorption spectrum of TiO_2_/PbS hybrid NCs (Figure 5A, blue line) spans over the UV-Vis-NIR spectral range. This confirms the presence of PbS domains. In particular, the spectrum shows a broad absorption at 545 nm (2.26 eV) consistent with the first exciton transition of small PbS NCs with sizes below 2 nm [73,74]. This is represented by the PbS domain decorating the TiO_2_ NCs and a week band at 850 nm (1.46 eV) ascribed to exciton transition of homogeneously nucleated PbS NCs, with a size of nearly 3 nm, present in the sample at a very low extent. The band broadening can be attributed to the broad size distribution of PbS domains, which is also revealed by the TEM characterization (Figure 2E). 

The PL spectra in the visible region reported in Figure 5B (λ_ex_= 375 nm) show a structured emission with two main bands peaked at nearly 415 nm and 435 nm for both samples and an emission tail extending up to 600 nm and 700 nm for the TiO_2_ and TiO_2_/PbS hybrid NCs, respectively. However, an increased intensity, without evident modifications of the line profile, is detected for the emission spectrum of the TiO_2_/PbS hybrid structures compared to the TiO_2_ NC seeds. 

Gaussian deconvolution (Figure S4A,B) of the PL spectrum of each sample (TiO_2_ NC, Figure S4A and TiO_2_/PbS hybrid NC Figure S4B) performed using four components results in emission bands centered at 411 nm (3 eV), 434 nm (2.86 eV), 445 nm (2,78 eV), and 462 nm (2.68 eV) for the TiO_2_ NC sample and at 413 nm (3 eV), 439 nm (2.82 eV), 446 nm (2.78 eV), and 488 nm (2.5 eV) for the TiO_2_/PbS sample, respectively. The component centered at 462 nm, for the TiO_2_ NC sample, and 488 nm, for the TiO_2_/PbS sample, are the one that is mainly contributed to the emission tail in the green-red visible spectral region. Early studies [75] attribute blue emission (400–450 nm) in TiO_2_ NCs to self-trapped exciton (STE), which corresponds to self-localized photogenerated charges, that, in anatase TiO_2_, can be more easily formed thanks to the long Ti-Ti interionic distances, the low TiO_6_ octahedral coordination, and the limited symmetry of the structure [75].

Emissive bands mainly located in the green and red spectral region are attributed to structural defects like Ti-interstitials and/or oxygen vacancies, which introduce sub bandgap defect states [76] that contribute to recombination paths of photogenerated electrons with holes trapped on undercoordinated Ti^3+^ or involve shallow states. Even though these emission bands become particularly relevant in high surface area materials such as NCs [75], which are expected to feature a high density of trap/defect states, the PL spectra of the synthesized TiO_2_ and TiO_2_/PbS hybrid NCs show only an emission tail in the green and red spectral region. This result can be explained by considering that oxygen vacancies suffer from the presence of O_2_ when acting as a scavenger of conduction band electrons and induce a strong PL quenching [71,75]. 

On the basis of these considerations, the fitting contributions of the TiO_2_ NC PL band centered in the range of 400–450 nm can be mainly attributed to STE while the one centered at 462 nm is ascribed to indirect recombination via oxygen defects [77]. The week emission of the TiO_2_ NC sample with the tail almost completely quenched at 600 nm suggests a low density of oxygen defects compared to the TiO_2_/PbS hybrid nanostructure. In the TiO_2_/PbS hybrid sample, a broad and more intense emissive component centered at 488 nm (2.6 eV) and extending up to 700 nm is displayed. This band is attributed to the charge transfer from Ti^3+^ to the nearby oxygen anion in a TiO_6_^8−^ complex structure, and also most likely originated from the recombination of electrons at oxygen-related defect states with the holes in the valence band [71]. Since oxygen vacancies improve the formation of STE, an increased intensity of the emission in the blue spectral region is detected for the TiO_2_/PbS hybrid sample. The increase in the surface oxygen vacancy states is also corroborated by the decrease of the average lifetimes of the decay profile. A faster PL decay (Figure S3) at 460 nm has been also measured with averaged lifetimes decreasing from 23 ns for TiO_2_ seeds to only 5 ns in the hybrid structures, which indicates the introduction of non-radiative pathways in the recombination of the excited charge carriers. Since oxygen vacancies are more stable, they do not remain as surface defect states, but tend to migrate at subsurface layers, which affects the TiO_2_ structures and potentially generates a partially amorphous layer [71], as corroborated by the XRD characterization. 

Only a very weak emission of the PbS domain has been observed. The lack of a significant emission signal in the far visible range expected for the PbS domain of 2 nm could be possibly ascribed to a PL quenching induced by electron transfers from PbS to TiO_2_ or to a strong red shift of the PL due to energy transfer phenomena among PbS NCs in close proximity with each other. A week and broad band, located in the NIR region at 1095 nm (1.14 eV), is displayed in Figure S4C. However, more in-depth characterization needs to be carried out to confirm the charge transfer process at the interface.

## 3. Materials and Methods 

### 3.1. Materials

Titanium(IV) ethoxide (TEO, technical grade), 1-decanol (DL, ~97%), 1-octadecene (ODE, technical grade 90%)), oleic acid (OA, technical grade 90%), oleylamine (Olam, technical grade 70%), lead oxide (II) (PbO, ≥99.0%), and hexamethyldisilathiane (HMDS, synthesis grade) were used for NCs synthesis. Acetone (≥99.5%) and ethanol (≥99.8%) were used as non-solvents to recover the colloidal NCs from the reaction mixture. Hexane and tetrachloroethylene (TCE, A.C.S. spectrophotometric grade ≥99%) were used as solvents to disperse the synthesized NCs. All reagents and solvents were purchased by Sigma-Aldrich (Milan, Italy) and used without further purification.

### 3.2. Synthesis of Organic-Capped TiO_2_ NCs

1 mmol of OA and 13 mmol of DL were dissolved in 15 mL of ODE and degassed for 1 h at 120 °C in a three-necked flask. Then, the temperature was set to 290 °C and 1 mmol of TEO was rapidly injected. The reaction was stopped after 1 h by cooling the reaction flask to room temperature. TiO_2_ NCs were collected by adding acetone and three cycles of centrifugation/re-dispersion in hexane with the addition of acetone. NCs were dispersed in hexane (OA-capped TiO_2_ NCs). Olam-capped TiO_2_ NCs were prepared by a ligand exchange reaction on OA-capped TiO_2_ NCs with Olam as follows: 1.5 mL of extensively washed native OA-capped TiO_2_ NCs were dispersed in 10 mL of ODE in the presence of 0.6 mmol of Olam and sonicated for more than 1 h. The colloidal solution was purified by non-solvent addition, centrifugation, and re-dispersion in hexane.

### 3.3. Synthesis of TiO_2_/PbS Hybrid NPs

The TiO_2_/PbS NC hybrid structures were synthesized by a seed-mediated growth reaction. In a three-neck flask, 1.5 mL of TiO_2_ NC seeds, 0.2 mmol, (either OA-capped or Olam-capped TiO_2_) were dispersed in 10 mL of ODE and degassed at 80 °C for 30 min. In another flask, 0.2 mmol of PbO were dispersed in 10 mL of ODE in the presence of 0.6 mmol OA under inert atmosphere and heated up to 120 °C for 30 min to decompose the PbO. The Pb-oleate precursor was then injected in the flask with the TiO_2_ NCs’ colloidal dispersion and was stirred under nitrogen for 10 min ([Pb^2+^]= 0.01 M ÷ 0.005 M), which was followed by the injection of HMDS sulfur precursor (HMDS: Pb molar ratio 1:5) at the different injection temperatures: 120 °C, 100 °C, and 80 °C (T_inj_) and the sudden decrease of the temperature down to 80 °C (growth temperature T_growth_) to stop the nucleation. The reaction mixture was left to stir for 10 min and then stopped by cooling down the solution. The NPs were collected by the addition of ethanol as a non-solvent and washed with three cycles of centrifugation/re-dispersion in hexane. The samples were then re-dispersed in 2 mL of TCE for further characterization. Three batches have been prepared for each synthetic approach in order to confirm the reproducibility of the morphological and spectroscopic results.

### 3.4. Sample Characterization 

UV-Vis-NIR absorption spectra were recorded by using a Cary 5000 spectrophotometer (Varian, Agilent Technologies Italia S.p.A, Milano, Italy). Steady-state and time-resolved UV−Vis-NIR photoluminescence experiments (PL) were performed by using a Fluorolog 3 spectrofluorimeter (Horiba Jobin-Yvon, Roma, Italy) equipped with both a continuous wave Xe lamp (450 W) and a ∼80 ps pulsed laser source (NanoLED 375 L), which emitted at 375 nm with a repetition rate of 1 MHz, and interfaced with a TBX-PS photon counter for steady-state and time-resolved (TRPL) measurements in the visible range and with a Peltier-cooled InGaAs detector for the NIR range. Fast Fourier transform infrared (FTIR) spectroscopy measurements were carried out in attenuated total reflection (ATR) mode with Spectrum One FTIR spectrometer (Perkin–Elmer, Milan, Italy) equipped with a triglycine sulfate (TGS) detector. The spectral resolution was 4 cm–1. The internal reflection element (IRE) was a three bounce 4 mm diameter diamond microprism. Cast films were prepared directly onto the IRE by depositing the sample solutions (3–5 lL) onto the upper face of the diamond crystal and allowing the solvent to evaporate. For morphological characterization, a JEM-1011 transmission electron microscope (TEM) of JEOL (Tokyo, Japan) was employed, operating at 100 kV acceleration voltage. Samples were prepared by dipping the carbon-coated copper grid in the colloidal dispersion of the NCs and NPs, prepared at a suitable dilution, and let the solvent evaporate. The particle average size and size distribution was obtained by counting at least 150 particles for each sample by means of a freeware Zeiss AxioVision analysis program (Jena, Germany). In particular, the average size was measured and the percentage relative standard deviation (σ%) was calculated in order to define the NC size distributions. High-angle annular dark field-scanning TEM (HAADF-STEM) images and energy-dispersive X-ray spectroscopy (EDS) analyses were acquired using an image C_s_-corrected JEM-2200FS TEM (JEOL) with a Schottky emitter, operated at 200 kV, equipped with a Bruker (Berlin, Germany) Quantax 400 STEM system and a XFlash 5060 silicon-drift detector (60 mm^2^ active area). The EDS spectra were quantified by the Cliff-Lorimer method applied to the O Kα peak (at 0.52 keV), the S Kα peak (at 2.31 keV), and the Pb Lα peak (at 10.55 keV). For these analyses, the samples were prepared by drop-casting the colloidal suspensions onto a double amorphous carbon film (ultrathin on holey)-coated Cu grid. X-ray photoelectron spectroscopy (XPS) characterizations were performed on an Axis UltraDLD spectrometer (Kratos) using a monochromatic Al Kα source (15 kV, 20 mA). The binding energy was calibrated by setting the main C1*s* peak (corresponding to C–C bonds) to 284.8 eV. A D8 Discover X-ray powder diffractometer (Bruker AXS Advanced X-ray Solutions GmbH, Karlsruhe, Germany) was used in Bragg-Brentano θ/2θ acquisition geometry using a copper Kα x-ray tube (0.154 nm) and a scintillation detector. The XRD patterns were recorded at a fixed incidence angle of 5° while moving the detector in the range 10–120° with a step size of 0.05°. A qualitative analysis of the crystalline phase content was performed using the QUALX 2.0 program [78]. Samples for XRD characterizations were prepared by drop casting of concentrated NPs’ dispersions on silicon substrates.

## 4. Conclusions

A seeded growth combined with a hot-injection approach has been used to prepare TiO_2_/PbS hybrid structures under a controlled experimental condition (reaction mixture composition, seed surface chemistry, and injection/nucleation temperature), suitably defined to limit homogenous nucleation and favor heterogeneous nucleation. The morphological and chemical characterization confirms the formation of small dots ascribed to PbS NCs decorating the oleylamine capped TiO_2_ NCs, upon injection at 100 °C of the Pb and S precursors, at the concentrations of 0.005 M and 0.001 M, respectively. The hybrid structure topology has been demonstrated to be strongly affected by the high interfacial strain between the TiO_2_ and PbS, which agreed with the Volmer-Weber growth mode. 

The XRD characterization suggests a structural modification of the TiO_2_ seeds in the hybrid structure with partial disruption of the TiO_2_ anatase crystallinity, likely induced to accommodate the misfit between the two materials’ lattice parameters and reduce the interfacial strain. The presence of dominant defect states in the TiO_2_ domain of the hybrid structure is confirmed by steady state and time resolved photoluminescence. The enhanced intensity of the emission band for the TiO_2_/PbS hybrid NCs compared to TiO_2_ NCs has been ascribed to a higher density of defect oxygen vacancies with emissive states in the green region and improved formation of STE whose emission falls in the blue region of the visible spectrum. 

## Figures and Tables

**Table 1:** STEM-EDS analysis (atomic %) of TiO_2_ NCs and TiO_2_/PbS hybrid structure.

	Ti%	O%	Pb%	S%
TiO_2_ NCs	33	66	-	-
TiO_2_/PbS NCs	20	73	8	low

## Supplementary Materials

The following are available online. Figure S1: FTIR spectra of neat oleic acid and oleylamine. Figure S2: TEM micrograph of oleic acid-capped TiO_2_ NCs after injection of [Pb^2+^] = 0.01M, [HMDS] = 0.002 M at 100 °C. Figure S3: Time-resolved photoluminescence decay at 460 nm (λ_ex_ = 375 nm) of TiO_2_ NCs and PbS/TiO_2_ hybrid nanostructures. Figure S4: Gaussian deconvolution of photoluminescence spectra (λ_ex_ = 375 nm) in the visible range and in the NIR range of TiO_2_ NCs and PbS/TiO_2_ hybrid nanostructures.
