# Supplementary material for: PbS Quantum Dots Decorating TiO2 Nanocrystals: Synthesis, Topology, and Optical Properties of the Colloidal Hybrid Architecture
Source: Molecules. 2020 Jun 26;25(12):2939. doi: 10.3390/molecules25122939 (PMC7356616; doi:10.3390/molecules25122939)

molecules
MDPI

Supplementary Information

PbS Quantum Dots Decorating TiO_2 Nanocrystals: Synthesis, Topology, and Optical Properties of the Colloidal Hybrid Architecture

Carlo Nazareno Dibenedetto [1,2], Teresa Sibillano [3], Rosaria Brescia [4], Mirko Prato [4], Leonardo Triggiani [2], Cinzia Giannini [3], Annamaria Panniello [2], Michela Corricelli [1], Roberto Comparelli [2], Chiara Ingrosso [2], Nicoletta Depalo [2], Angela Agostiano [1,2], Maria Lucia Curri [1,2], Marinella Striccoli [2,*] and Elisabetta Fanizza [1,2,*]

[1] Dipartimento di Chimica, Università degli Studi di Bari, Via Orabona 4, 70126 Bari, Italy; carlo.dibenedetto@uniba.it (C.N.D); michela.corricelli@gmail.com (M.C.); angela.agostiano@uniba.it (A.A); marialucia.curri@uniba.it (M.L.C.)
[2] CNR-Istituto per i Processi chimico Fisici (CNR-IPCF), SS Bari, Via Orabona 4, 70126 Bari, Italy; l.triggiani@ba.ipcf.cnr.it (L.T.); a.panniello@ba.ipcf.cnr.it (A.P.); r.comparelli@ba.ipcf.cnr.it (R.C.); c.ingrosso@ba.ipcf.cnr.it (C.I.); n.depalo@ba.ipcf.cnr.it (N.D.)
[3] CNR-Istituto di Cristallografia (CNR-IC, Via Amendola, 122/O, 70126 Bari, Italy; teresa.sibillano@ic.cnr.it (T.S.); cinzia.giannini@ic.cnr.it (C.G.)
[4] IIT- Istituto Italiano di Tecnologia, via Morego 30, 16163 Genova, Italy, Rosaria.Brescia@iit.it (R.B.); Mirko.Prato@iit.it (M.P.)
* Correspondence: m.striccoli@ba.ipcf.cnr.it (M.S.); elisabetta.fanizza@uniba.it (E.F.); Tel: +39-080-544-2027 (M.S. & E.F.)

Academic Editor: Giuseppe Cirillo
Received: 11 June 2020; Accepted: 24 June 2020; Published: 26 June 2020

Figure S1. FTIR spectra in ATR mode of neat oleic acid (a, a1, black line) and oleylamine (b, b1, red line).

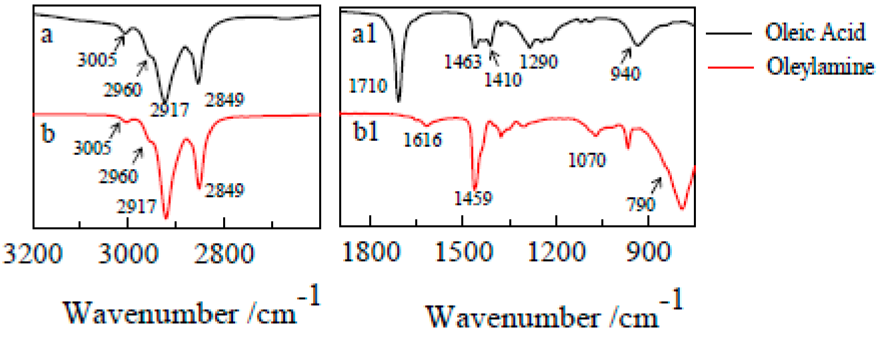

Figure S2. TEM micrograph (scale bar 20 nm) of oleic acid (OA)-capped TiO2 NCs, after injection of [Pb2+] = 0.01M, [HMDS] = 0.002 M at 100°C.

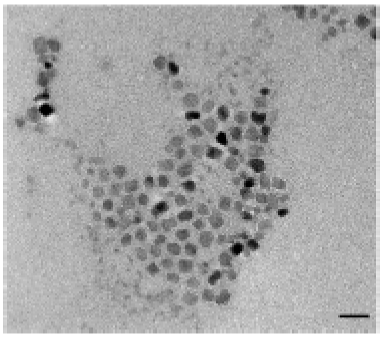

Figure S3. Time-resolved PL decay at 460 nm (λex= 375 nm) of oleylamine-capped TiO2 NCs (red line) and TiO2/PbS nanostructure (blue line) and fitting parameters of the PL decay profiles fitted by three exponentials. The terms B1%, B2%, B3% represent the weight percentual of the three times #**τ** used for the fitting.

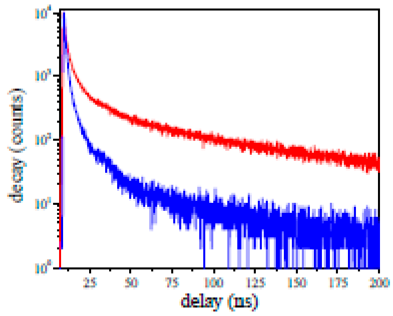

	B1%	B2%	B3%	τ1 (ns)	τ2 (ns)	τ3 (ns)	τAV (ns)
TiO_2	25.7	29.6	44.8	1.3	6.6	55.3	27.0
TiO2/PbS	55.6	32.1	12.3	0.9	3.8	26.3	5.0

Figure S4. Gaussian deconvolution of photoluminescence spectra (位 ex= 375 nm) reported in Figure 5 in the main paper of oleylamine-capped TiO2 NCs (A) and TiO2/PbS hybrid structures (B). The deconvolution procedure reveals that both the spectra are mainly composed by four peaks centred at 411 nm (3 eV), 434 nm (2.86 eV), 445 nm (2,78 eV) and 462 nm (2.68 eV) for the TiO2 NCs sample and at 413 nm (3 eV), 439 nm (2.82 eV) 446 nm (2.78 eV) and 488 nm (2.5 eV) for the TiO2/PbS NCs nanostructures respectively, arising from different radiative transitions. (C) NIR emission ascribed to homogeneously nucleated PbS.

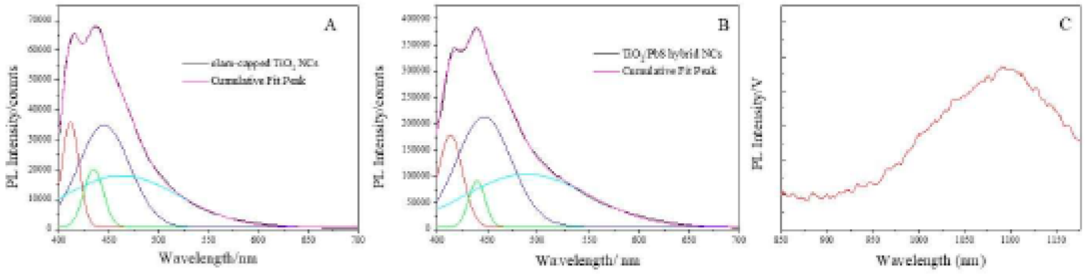

Supplement: Supplementary file 1 [file molecules-25-02939-s001.pdf]
